# The Association of Axonal Damage Biomarkers and Osteopontin at Diagnosis Could Be Useful in Newly Diagnosed MS Patients

**DOI:** 10.3390/neurolint17070110

**Published:** 2025-07-17

**Authors:** Eleonora Virgilio, Chiara Puricelli, Nausicaa Clemente, Valentina Ciampana, Ylenia Imperatore, Simona Perga, Sveva Stangalini, Elena Boggio, Alice Appiani, Casimiro Luca Gigliotti, Umberto Dianzani, Cristoforo Comi, Domizia Vecchio

**Affiliations:** 1 Department of Clinical and Biological Sciences, University of Turin, 10100 Turin, Italy; 2 Interdisciplinary Research Center of Autoimmune Diseases (IRCAD), Department of Health Sciences, University of Piemonte Orientale, Corso Mazzini 18, 28100 Novara, Italy; nausicaa.clemente@med.uniupo.it (N.C.); umberto.dianzani@maggioreosp.novara.it (U.D.); cristoforo.comi@med.uniupo.it (C.C.); domizia.vecchio@gmail.com (D.V.); 3 Clinical Biochemistry, University Hospital Maggiore della Carità di Novara, 28100 Novara, Italy; 20032501@studenti.uniupo.it (C.P.); simonaperga77@gmail.com (S.P.); sveva.stangalini@maggioreosp.novara.it (S.S.); alice.appiani@maggioreosp.novara.it (A.A.); 4 Neurology Unit, Department of Translational Medicine, University of Piemonte Orientale, Maggiore della Carità University-Hospital, 28100 Novara, Italy; 20022116@studenti.uniupo.it (V.C.); 20042798@studenti.uniupo.it (Y.I.); 5 Department of Health Sciences, University of Piemonte Orientale, 28100 Novara, Italy; elena.boggio@med.uniupo.it (E.B.); luca.gigliotti@med.uniupo.it (C.L.G.)

**Keywords:** multiple sclerosis, biomarker, osteopontin, neurofilament, neurodegeneration, tau

## Abstract

(1) Background: Multiple sclerosis (MS) is a biologically highly heterogeneous disease and has poor predictability at diagnosis. Moreover, robust data indicate that early disease activity strongly correlates with future disability. Therefore, there is a need for strong and reliable biomarkers from diagnosis to characterize and identify patients who require highly effective disease-modifying treatments (DMTs). Several biomarkers are promising, particularly neurofilament light chains (NFLs), but the relevance of others is less consolidated. (2) Methods: We evaluated a panel of axonal damage and inflammatory biomarkers in cerebrospinal fluid (CSF) and matched serum obtained from a cohort of 60 newly diagnosed MS patients. Disability at diagnosis, negative prognostic factors, and the initial DMT prescribed were carefully recorded. (3) Results: We observed correlations between different axonal biomarkers: CSF and serum NFL versus CSF total tau; and between the inflammatory marker osteopontin (OPN) and axonal biomarkers CSF p-Tau, CSF total tau, and serum NFL. CSF and serum NFL and total tau, as well as CSF OPN, positively correlated with EDSS at diagnosis. Moreover, CSF and serum NFL levels were increased in patients with gadolinium-enhancing lesions (*p* = 0.01 and *p* = 0.04, respectively) and in those treated with highly effective DMT (*p* = 0.049). Furthermore, CSF OPN and both CSF and serum NFL levels significantly differentiated patients based on EDSS, with a combined ROC AUC of 0.88. We calculated and internally validated biomarker (in particular serum NFL) thresholds that significantly identified patients with higher disability. Finally, CSF OPN levels and dissemination in the spinal cord were significant predictors of EDSS at diagnosis. (4) Conclusions: These preliminary exploratory data confirm the pathological interconnection between inflammation and axonal damage from early disease stages, contributing to early disability. Follow-up data, such as longitudinal disability scores, repeated serum measurements, a healthy control group, and external validation of our results, are needed. We suggest that combining several fluid biomarkers may improve the clinical characterization of patients.

## 1. Introduction

Multiple sclerosis (MS) is a highly heterogeneous autoimmune disease. This disorder is characterized by the accumulation of disability through both acute and chronic inflammation, which can be compartmentalized. In addition, neuronal loss from disease onset onward could be the result of both focal and diffuse damage [1]. MS patients may experience both relapse-associated worsening and progression independent from relapse activity, which are both present from the early stages of the disease [2]. However, the disease course, rate of disability accumulation, and treatment response are highly variable. Moreover, multiple disease-modifying therapies (DMTs) are currently available [3,4]. Numerous data support an induction approach that utilizes highly effective treatments from early stages to achieve remission of disease activity [5]. However, the first treatment choice is still based on demographic, clinical, and radiological characteristics to distinguish between negative prognostic outcomes and a possibly milder evolution [5,6]. Finally, an escalation approach starting with low-efficacy treatment in patients with few negative prognostic factors is still used [7]. Highly effective treatments are associated with an increased risk of side effects; moreover, little is known about their efficacy in acting on the compartmentalization of smoldering inflammation, microglial activation, and neurodegeneration [8]. Fluid biomarkers could help to partially unravel these mechanisms and direct the first therapeutic decision [9,10,11]. In clinical practice, oligoclonal band status serves as a diagnostic and prognostic biomarker in cerebrospinal fluid (CSF), since the presence of oligoclonal bands is a negative prognostic factor. However, only 10–20% of patients are oligoclonal band-negative [12]. Other fluid biomarkers may play a key role in the clinical management of MS, supporting diagnostic and prognostic purposes and treatment decision-making [9]. Neurofilament light chains (NFLs) are structural proteins of the neuronal cytoskeleton and were extensively studied in MS [10,13,14]. CSF NFLs are linked to relapses and MRI activity and might predict disease worsening [14,15,16]. Moreover, recent sensitive methodologies opened the way to use NFL levels also in the serum [15] as detected using single-molecule assay (SIMOA™) or other platforms, including the Simple Plex Human NF-L Cartridge on the ELLA microfluidic platform [17,18]. A second structural protein released upon neuronal damage is total tau (t-Tau), which is essential for microtubule stabilization, neuronal dynamics, and axonal transport [19]. Moreover, multiple studies have shown a connection between microglial activation and tau protein [20] and investigated the potential of t-Tau as a biomarker in MS [19]. An increase in CSF t-Tau at MS diagnosis was previously reported in patients with higher disability over a mean follow-up of two years using ELISA. However, other studies have shown conflicting results in the MS population [21]. More sensitive technologies with good analytical performance have also been introduced, like the automated chemiluminescent enzyme immunoassay on the Lumipulse analyzer [22,23]. Moreover, some studies have also pointed out the possible use of phosphorylated tau (p-Tau) in addition to t-Tau, particularly in progressive MS patients with conflicting results [24,25]. Finally, there is an increasing interest in osteopontin (OPN), a multifunctional cytokine and adhesion protein that plays a key role in various physiological and pathological processes, including bone remodeling, tissue repair, tumor progression, vascular diseases, and inflammatory conditions [26]. OPN serves a pleiotropic role in neurological diseases, acting as a pro-inflammatory cytokine that contributes to neuroinflammation and is involved in both innate and adaptive responses, regulating microglia [27,28]. OPN expression increases in many autoimmune diseases, including MS [29]. OPN acts as both an immobilized extracellular matrix protein and a secreted free cytokine in body fluids, and several pieces of evidence support its detrimental role in the central nervous system [29]. During acute inflammation, OPN is cleaved into two fragments, binding α4β1 integrin, inducing interferon-γ production, and downregulating IL-10 [29]. Recently, some evidence correlated CSF OPN with progression independent from relapse activity and atrophy MRI markers in MS patients [30,31]. Ultimately, OPN could have a role in promoting neurodegeneration [30,31] by enhancing sustained microglial activation, inducing the release of neurotoxic molecules such as reactive oxygen species and pro-inflammatory cytokines, ultimately worsening neuronal damage [28]. Plus, several pieces of evidence support OPN secretion by multiple residence cells in the central nervous system, such as T cells and B cells, neutrophils, and microglia [27,32]. Microglia secrete osteopontin (OPN) into the extracellular matrix, promoting the activation and recruitment of macrophages and central nervous system resident cells, which in turn modulate the inflammatory response [30]. However, in the experimental animal model, OPN is thought to prevent neurodegeneration in motor neurons, likely through its regulation of matrix metalloproteinase expression, and to exert a promotive effect on axonal growth with the establishment of functional synapses within the central nervous system [33].

This work aimed to study the relationship between CSF and serum biomarkers representing different pathogenetic aspects in MS: axonal loss, microglial activation, and inflammation. We also aimed to investigate the relationship between our biomarkers and known MRI, demographic, and clinical predictors of worse prognosis at disease diagnosis. Finally, we evaluated the predictive value of our biomarkers in identifying patients with a worse disease course (higher EDSS and highly effective DMT start) from early disease stages.

## 2. Materials and Methods

### 2.1. Informed Consent

All patients signed an informed consent form at the time of the lumbar puncture. Our ethical committee approved the study on 27 January 2023 with the protocol number 65/CE (reference number CE 262/2022). All research followed relevant international guidelines/regulations.

### 2.2. Clinical Characteristics

We enrolled a cohort of 60 newly diagnosed, drug-naïve MS patients according to the revised McDonald Criteria of 2017 [34]. Disease onset was defined as the time of the first clinical manifestation attributable to MS, either corresponding to the symptom that prompted the first neurological evaluation or to a prior symptom retrospectively identified during the initial clinical history, for which no investigations had been performed at the time. Patients underwent a standard MS diagnostic work-up, including clinical evaluation, brain and spinal cord MRI, and lumbar puncture. The time of lumbar puncture, which coincided with the time of diagnosis, was considered the baseline for all enrolled patients. The lumbar puncture was performed before any DMT was started. We recorded the following clinical–demographic data: sex, age of onset, age at diagnosis, MS course, onset type, and EDSS at diagnosis. We analyzed MRI variables at diagnosis: white matter lesion load with a cut-off of 9 lesions [35,36], presence or absence of spinal cord lesions, and presence of at least one gd+ lesion. Brain and spinal MRIs were performed 6 months before or after baseline, as recommended by Italian guidelines [37]. We also recorded the exposure to the first DMTs, which started shortly after the diagnosis (within the first 6 months from baseline). Patients with optic neuritis (involving the intraorbital segment of the optic nerve) were diagnosed based on suggestive clinical presentation and paraclinical tests (altered automated visual field and visual evoked potential and/or MRI with gadolinium) [38]. Serum aquaporin4 and myelin oligodendrocyte glycoprotein antibodies were tested to rule out patients with neuromyelitis optica spectrum disorder and myelin oligodendrocyte glycoprotein-associated disorder. An extensive serologic rheumatologic screening was negative for autoimmune markers [38]. We excluded patients who had received steroids within 30 days prior to baseline, had ever been treated with immunosuppressive agents, lacked baseline serum or CSF samples, had a pediatric disease onset, or had other inflammatory conditions.

### 2.3. Laboratory Sampling

CSF specimens were collected through a lumbar puncture conducted between the L3 and L4 or L4 and L5 vertebral spaces at the time of diagnosis. Serum was collected contextually. Samples were divided into aliquots using polypropylene tubes and kept at −80 °C until needed. All experiments were performed on the same day, in the same laboratory by experienced operators to reduce batch effects. Both CSF and serum were thawed at 2–8 °C and kept at room temperature before being tested; we avoided repeated freeze–thaw cycles [39]. Patients are defined as newly diagnosed since lumbar puncture was performed shortly after the first neurological evaluation confirming the MS final diagnosis. Concentrations of t-Tau and p-Tau in CSF samples were measured using the automated chemiluminescent enzyme immunoassay method on the fully automated Lumipulse^®^ G600 II analyzer (Fujirebio Italia S.r.l., Pomezia, Rome, Italy), employing the specific Lumipulse^®^ G t-Tau and Lumipulse^®^ G pTau 181 Immunoreaction Cartridges, respectively [40,41]. To obtain the T-Tau calibration curve, 3 calibrators at 0 pg/mL, 582 pg/mL, and 2265 pg/mL were used, while for p-Tau, 3 calibrators at 0 pg/mL, 40 pg/mL, and 400 pg/mL were used. The producer-declared lower limit of quantitation was 141 pg/mL for t-Tau and 1.058 pg/mL for p-Tau. Before loading them on the automated analyzer, they were mixed by vortexing. Samples were used undiluted. Normative data (to detect cognitive impairment) are present for t-Tau and p-Tau with the Lumipulse^®^ G600 II. T-Tau is considered abnormal if higher than 410 pg/mL, and p-Tau is considered abnormal if higher than 60 pg/mL [41]. To measure the concentrations of NFL in CSF and serum samples, the Simple PlexTM fluorescence-based immunoassay by Bio-Techne was used on the Ella Simple PlexTM Platform (Bio-Techne s.r.l., Milan, Italy) [42]. Specifically, NFL was measured using the Human NFL Simple PlexTM Cartridge Kit. The factory-generated calibration curves were obtained by plotting the known concentration (average of 5 replicates) of 12 standards against the measured signal intensity in relative fluorescence units. For NFL, the lower limit of quantitation was 2.70 pg/mL, while the upper limit of quantitation was 10,290 pg/mL. CSF and serum samples, stored at −80 °C, were gradually thawed at 2–8 °C, equilibrated approximately to 20–25 °C, and centrifuged at 4000× *g* for 5 min prior to assay execution. For the NFL assay, CSF or serum samples were diluted 1:2 by mixing 50 µL of sample and 50 µL of sample diluent in each well of a 96-well plate [42]. No normative data are available for CSF and serum NFL on Ella Simple PlexTM Platform. Finally, OPN in both CSF and serum samples was determined on Simple Plex ELLA instrument (Bio-Techne). In particular, OPN was measured using the Simple PlexTM Cartridge Kit. The factory-generated calibration curve was compiled by averaging 5 replicates of each calibrator from multiple runs. This curve shows the calibrator concentration as a function of signal intensity. For OPN, the lower limit of quantitation was 20.97 pg/mL and the upper limit of quantitation was 12,800 pg/mL. The samples, CFS and serum, required dilution with sample diluent. No normative data are present for OPN.

### 2.4. Statistical Analysis

Results were analyzed using SPSS 25.0 (SPSS Inc., Chicago, IL, USA) and GraphPad Prism 9 (GraphPad Software, La Jolla, CA, USA). Normality was assessed via the Shapiro–Wilk test. Quantitative data are summarized using the mean and standard deviation, while qualitative data are described by the median, range, interquartile range, and frequencies with percentages. A comparison between groups was evaluated using the Mann–Whitney U test. Univariate analysis was performed with Spearman’s rank correlation coefficient test. ROC curves were computed to study each biomarker’s sensitivity and specificity and possibly identify a cut-off based on DMT treatment and disability evaluated with the EDSS. The Youden Index (computed as sensitivity + specificity − 1) was then calculated. Since we focused on early disability accumulation in the initial months following MS diagnosis, we used a cut-off of EDSS > 2.5. EDSS is not a linear scale, and previous studies have suggested that EDSS > 2.5 may serve as an early clinical threshold to stratify patients with a potentially increased risk of long-term disability and represents the critical transition from mild or no disability to moderate irreversible disability [43,44]. To internally validate our biomarker thresholds, we randomly split the dataset into a training subset (70% corresponding to 40 patients) and a validation subset (30% corresponding to 20 patients). ROC curve analysis and Youden’s index were re-run on the training dataset to confirm our results, and their diagnostic performance (sensitivity and specificity) was then evaluated in the independent validation subset.

Linear regression analyses were performed with biomarkers, MRI, and clinical characteristics at baseline as independent variables and EDSS or type of treatment as dependent variables. Alfa was set at <0.05.

## 3. Results

### 3.1. Baseline Characteristics

The main demographic features, clinical features, and disability scores at diagnosis are summarized in Table 1.

We enrolled a young cohort of patients (age at diagnosis 36.5 ± 10.7 years old) with a mean disease duration of 5 years (SD ± 7 years) from onset to diagnosis and a low median expanded disability status scale (EDSS) at diagnosis (median 1.5, IQR 1-2.5) (see Table 1). Fifty-three (89%) were relapsing–remitting MS (RRMS) patients, two (3%) had radiologically isolated syndrome, two (3%) had clinically isolated syndrome (CIS), and three (5%) had progressive MS (PMS). Clinically, seventeen (29%) patients presented optic neuritis as disease onset, sixteen (27%) presented a sensory or motor onset, fourteen (23%) presented a spinal onset, ten (16%) presented an infratentorial onset, and one (2%) patient demonstrated a multifocal onset. All the information is summarized in Table 1. Over half of the cohort displayed a high white matter lesion load, spinal cord involvement, and at least one gadolinium-enhancing (gd+) lesion. Mean concentrations of the included biomarkers at diagnosis were as follows: CSF t-Tau 215 pg/mL (SD ± 79.4 pg/mL; median: 201.5 pg/mL), CSF p-Tau 26.5 pg/mL (SD ± 7.9 pg/mL; median: 25.8 pg/mL), CSF NFL 2487 pg/mL (SD ± 4804 pg/mL; median: 1348 pg/mL), serum NFL 34.6 pg/mL (SD ± 24.6 pg/mL; median: 29.3 pg/mL) CSF OPN 174,013 pg/mL (SD ± 192,495 pg/mL; median: 105,455 pg/mL), and serum OPN 49,329 pg/mL (SD ± 30,418 pg/mL; median: 44,232 pg/mL). Only one patient displayed t-Tau above the normality cut-off (with a value of 530 pg/mL), and no patients displayed abnormal values of p-tau when applying the normative values. After diagnosis, based on prognostic clinical and radiological factors, thirty-eight patients (63%) started moderate-efficacy DMTs (interferons, glatiramer acetate, teriflunomide, dimethyl fumarate, and azathioprine), and sixteen patients (27%) started highly effective treatments (fingolimod, siponimod, natalizumab, alemtuzumab, cladribine, ocrelizumab and ofatumumab). Six patients (10%) did not start any DMT within six months from diagnosis: two CIS patients presented with optic neuritis and oligoclonal band as well as a normal brain and spinal MRI and were proposed either active moderate-efficacy DMT or active clinical–radiological follow-up and decided for the latter. One patient was diagnosed with non-active stable secondary progressive MS, and three RRMS patients were proposed moderate-efficacy DMTs but refused. Table 1 provides a schematic overview of all the features previously described.

### 3.2. Axonal and Inflammatory Biomarkers Positively Correlate at Diagnosis

Results are shown in Table 2.

We found a strong positive correlation between CSF t-Tau and p-Tau (rs: 0.76, *p* < 0.0001) and between CSF and serum NFL (rs: 0.8, *p* < 0.0001). Moreover, CSF and serum NFL moderately correlated with CSF t-Tau (rs: 0.45, *p* = 0.0004 and rs: 0.29, *p* = 0.02) but not with p-Tau. Serum and CSF OPN did not correlate with each other, but CSF OPN correlated with CSF p-Tau (rs: 0.30, *p* = 0.01) and t-Tau (rs: 0.26, *p* = 0.04), whereas serum OPN correlated with serum NFL (rs: 0.43, *p* = 0.0005).

### 3.3. Neurofilament Light Chains Are Higher in Patients with Acute Inflammation and High-Efficacy Treatments

When stratified by our baseline characteristics (shown in Table 3), we observed that patients with at least one gd+ lesion displayed higher CSF NFL and serum NFL than those lacking gd+ lesions (CSF: 3270 ± 5964 pg/mL vs. 1228 ± 994.7 pg/mL, *p* = 0.01; serum: 39.4 ± 29.2 pg/mL vs. 26.8 ± 11.3 pg/mL, *p* = 0.04). Moreover, patients treated with highly effective DMTs after diagnosis displayed higher CSF NFL than those treated with moderate-efficacy DMTs (4383 ± 8633 pg/mL vs. 1797 ± 1960 pg/mL, *p* = 0.049). When considering other radiological characteristics, no other statistically significant differences were observed.

### 3.4. High Neurofilaments and Osteopontin Predict Higher Disability at Diagnosis

CSF NFL, t-Tau, CSF OPN, and serum NFL positively correlated with EDSS at diagnosis (rs: 0.39, *p* = 0.001, rs: 0.27, *p* = 0.03, rs: 0.37, *p* = 0.0039 and rs: 0.24, *p* = 0.05).

This correlation was also confirmed after correction for age and sex as covariates (rs: 0.47, *p*: 0.001; rs: 0.35, *p*: 0.006; rs: 0.25, *p*: 0.048; rs: 0.43, *p*: 0.001).

**Figure 1:**
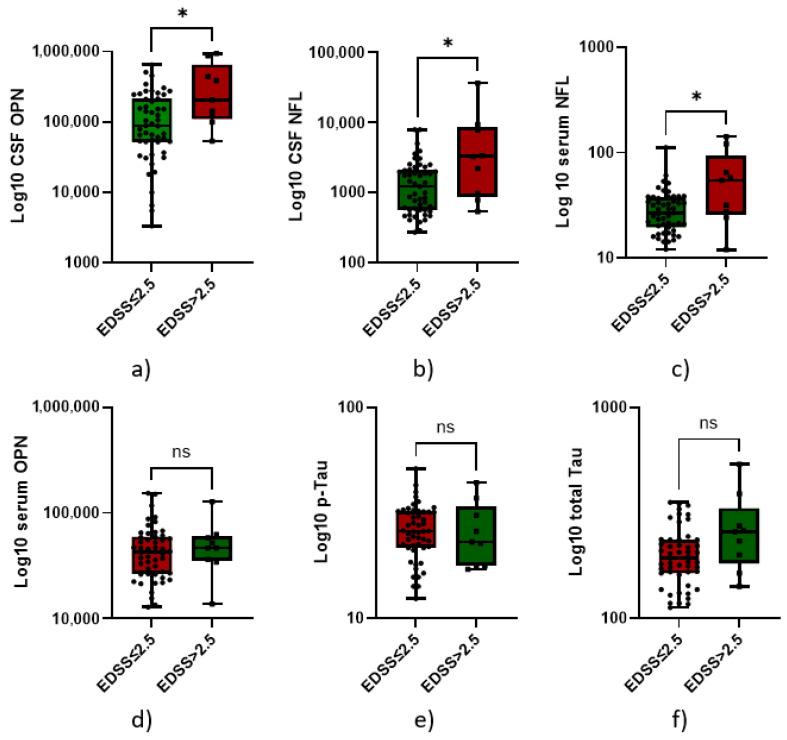
Box plots comparing biomarker levels (expressed in Log10) in patients with multiple sclerosis (MS) stratified by expanded disability status scale (EDSS) scores at diagnosis using a cut-off of 2.5. Each subplot illustrates the distribution of a specific biomarker in cerebrospinal fluid (CSF) or serum: (**a**) CSF osteopontin (OPN) levels are significantly elevated in patients with EDSS > 2.5 compared to those with EDSS ≤ 2.5 (*p* < 0.05). (**b**) CSF neurofilament light chain (NFL) levels are also significantly higher in the EDSS > 2.5 group (*p* < 0.05). (**c**) Serum NFL concentrations show a similar pattern, with significantly increased levels in the EDSS > 2.5 group (*p* < 0.05). (**d**) Phosphorylated tau (p-Tau) levels in CSF do not significantly differ between the two groups (ns = not significant). (**e**) Serum OPN levels also show no significant difference between the EDSS categories. (**f**) Total tau (t-Tau) levels in CSF are not significantly different between groups. Statistical significance: *p*  <  0.05 is indicated by an asterisk (*); ns = not significant.

In contrast, p-Tau and serum OPN did not. Patients with EDSS > 2.5 displayed significantly higher levels of CSF NFL (1673 ± 1629 pg/mL vs. 7097 ± 11,202 pg/mL, *p* = 0.02), serum NFL (30.16 ± 16.22 pg/mL vs. 59.57 ± 44.61 pg/mL, *p* = 0.04), and CSF OPN (141,096 ± 136,684 pg/mL vs. 360,541 ± 332,453 pg/mL, *p* = 0.02) than patients with EDSS < 2.5, whereas no differences were found for CSF t-Tau (*p* = 0.07), CSF p-tau (*p* = 0.7), and serum OPN (*p* = 0.5) (Figure 1).

**Figure 2:**
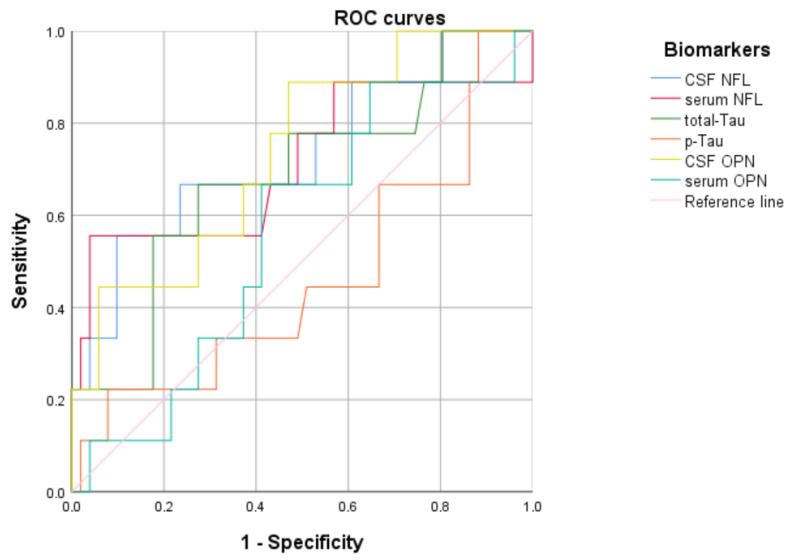
ROC curves computed for each biomarker based on disability evaluated with the EDSS (using a cut-off of 2.5). Significant results (*p* < 0.05) with an AUC between 0.7 and 0.8 were obtained for cerebrospinal fluid and serum neurofilament light chain and cerebrospinal fluid osteopontin.

The receiver operating characteristic (ROC) analysis for each biomarker and EDSS (using 2.5 as a cut-off) showed significant results for CSF NFL (*p* = 0.02), with an area under the curve (AUC) of 0.73 with a cut-off of 3159 pg/mL (youden index of 0.458); CSF OPN (*p* = 0.02), with an AUC of 0.73 and a cut-off of 96,846.5 pg/mL (youden index of 0.42); and serum NFL (*p* = 0.04), with an AUC of 0.71 and a cut-off of 54.1 pg/mL (youden index of 0.516) (Figure 2).

**Figure 3:**
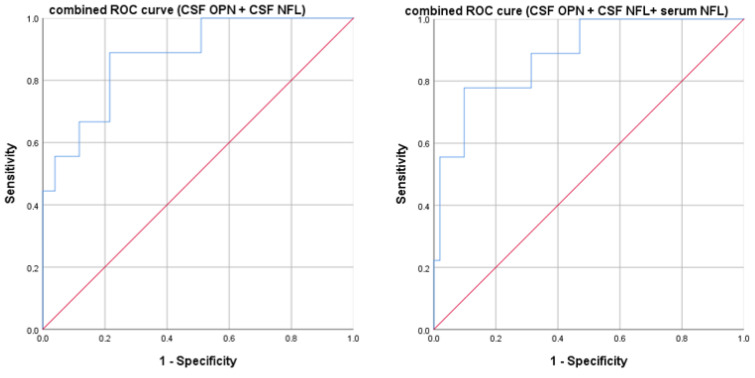
Multivariate ROC curves computed for combined biomarkers based on disability evaluated with the EDSS using a cut-off of 2.5. Both the models were statistically significant (*p* < 0.05). On the left, the model with cerebrospinal fluid osteopontin and neurofilament light has an AUC of 0.878; on the right is the same model after adding serum neurofilament light chains, with an AUC of 0.885.

We then performed a multivariate ROC analysis combining the significant biomarkers: CSF OPN and CSF NFL showed an AUC of 0.878 (*p* < 0.0001). By adding serum NFL to the other two markers, we obtained an AUC of 0.885 (*p* < 0.0001). Results are graphically reported in Figure 3.

Finally, to internally validate our biomarker thresholds, we randomly split the dataset into a training subset (70%) and a validation subset (30%). We confirmed in the training subset the significant results with high AUC for CSN NFL, CSF OPN, and serum NFL (Table S1, Supplementary Materials). We confirmed the same cut-off for all three biomarkers (and similar Youden indexes, reported in Supplementary Materials). We then proceeded to apply our thresholds in the remaining validation dataset (corresponding to 30% of our total, randomly generated). In the validation subset, only one patient out of 20 showed an EDSS score > 2.5. The previously calculated CSF NFL threshold showed a high specificity (94.7%) but failed in identifying the patient with an EDSS score > 2.5, resulting in an overall accuracy of 90%. The serum NFL cut-off identified in the training set showed excellent diagnostic performance when applied to the independent test subset. It reached a sensitivity of 100%, a specificity of 94.7%, and an overall accuracy of 95%. Finally, in the independent test set, the threshold identified for CSF OPN yielded a sensitivity of 100%, a specificity of 52.6%, with an overall accuracy of 55%.

No differences in biomarkers were observed between different types of onset, even if patients with cerebellar and spinal cord onset displayed higher EDSS scores compared to other categories (mean EDSS 2.3 ± 1.3 for cerebellar/brainstem vs. 2.3 ± 0.9 for spinal vs. 1.8 ± 1.6 for sensory/motor vs. 1 ± 0.7 for optic neuritis vs. 0 ± 0 for radiologically isolated syndrome and 2 for the patient with multifocal onset, *p* = 0.006). A trend for higher t-Tau (*p* = 0.2), p-tau (*p* = 0.08), CSF NFL (*p* = 0.7), and serum OPN (*p* = 0.3) was observed in male patients compared to females, but this was statistically significant only for CSF OPN levels (230,248 ± 210,723 in males vs. 141,455 ± 175,827 in females, *p* = 0.01). Age at diagnosis did not correlate with our biomarkers except for CSF OPN (rs: 0.35, *p* = 0.007). Finally, when performing a linear regression analysis with EDSS as the dependent variable and demographic (age and sex), clinical, and radiological parameters and biomarkers as independent variables, we obtained a significant model (r2 adjusted 0.609, *p* < 0.0001) with CSF OPN, disease duration, and spinal cord lesions being the two significant predictors (see Table S2, Supplementary Materials).

### 3.5. Cerebrospinal Fluid Neurofilament and First DMT Choices

We then evaluated whether our biomarkers could differentiate patients based on the first DMT received. When performing a ROC analysis for each biomarker and the type of first DMT choice, only CSF NFL was significant (*p* = 0.04) with an AUC of 0.66, indicating limited clinical use. However, our cohort demonstrated that patients with CSF NFL above 1274 pg/mL (and Youden index 0.318) are more likely to be treated with highly effective first treatments based on negative prognostic, clinical, demographic, and radiological factors at diagnosis. We again validated these results internally, using both the training and validation datasets. However, most biomarkers demonstrated poor to modest discriminatory power in the training dataset. CSF-NFL and serum-NFL reached AUC values of 0.662 and 0.652, respectively, but neither achieved statistical significance (*p* = 0.097 and *p* = 0.118). Other biomarkers such as tau, p-tau, and osteopontin (both serum and CSF) had AUCs close to or below 0.5, indicating no relevant discriminative capacity in this setting. Finally, when performing a linear regression analysis, using the treatment choice as a dependent variable and demographic (age and sex) and radiological data and biomarkers as independent variables, we obtained a significant model (r2 adjusted of 0.348 and *p* 0.001) with white matter lesion load and EDSS being the significant predictors (see Table S3 of Supplementary Materials).

## 4. Discussion

Our study supports the hypothesis that early disability at MS diagnosis is a result of both inflammation (which we related to OPN) and axonal loss (expressed by NFL level and t-Tau), possibly due to both acute activity and smoldering disease, as observed in Figure 1 [8]. Several cell types (T and B cells, macrophages, neutrophils, and dendritic cells) in the central nervous system express OPN, supporting cell migration and proliferation, immune response, and inflammatory processes [32]. Several previous studies have shown that elevated serum NFL is linked to both current disability progression and, to a lesser extent, future disability accumulation [45]. Moreover, at diagnosis, we observed that a combination of several different biomarkers (CSF and serum NFL along with CSF OPN) was able to differentiate patients with a higher EDSS, helping to characterize and identify those with a high risk of confirmed disability, as observed in Figure 3 and the Supplementary Materials. This is fundamental to correctly choose the first DMT, since the first-year treatment response predicts the following five-year disease course and irreversible disability accumulation [46]. However, highly effective DMT could be associated with severe side effects; therefore, the presence of early prognostic biomarkers could help the first therapeutic choice. The internal cross-validation confirmed the same cut-offs in the randomly generated training dataset. However, when applying them, diagnostic accuracy in the validation set was optimal for serum NFL, supporting the robustness of the 54.1 pg/mL cut-off for identifying patients with an EDSS score > 2.5. In contrast, for CSF NFL, although the cut-off was effective in ruling out patients with low EDSS scores, it failed to detect those with higher disability. This result could be due to the small sample size. Finally, regarding CSF OPN, although the cut-off was effective in identifying patients with EDSS scores > 2.5, it came at the cost of a substantial number of false positives among those with lower disability. Since external validation on a different dataset is currently lacking, our results need to be replicated and validated in a larger future cohort.

We found a strong positive correlation between different biomarkers of axonal damage, particularly between NFL and t-Tau, further supporting that axonal damage involves all the byproducts of neurons (reported in Table 2). Moreover, recent evidence also supports the pathogenetic role of tau in MS since t-Tau has been found along with p-Tau inside and close to chronic active lesions in human MS brain tissues of progressive patients and experimental animal models [20]. In our experiment, p-Tau seemed less informative than t-Tau, but this might be related to the small sample size of our progressive MS patients. Furthermore, we showed that NFL are increased in both serum and CSF, are higher in acute inflammation (represented by at least one gd+ lesion−Table 3), suggesting that NFL might be used to track acute axonal damage secondary to inflammation, whereas t-Tau might track chronic and smoldering axonal loss [21,47].

Interestingly, our experiment confirms previous results regarding the correlation between CSF OPN and NFL [48] and the absence of correlation between CSF and serum OPN (Table 2) [49]. These results support the hypothesis that CSF OPN may reflect intrathecal inflammatory activity, potentially involving microglial activation among other immune-mediated mechanisms within the central nervous system. In the literature, OPN was proven to have both protective and harmful effects, promoting tissue repair in acute injuries like stroke, while potentially exacerbating damage in chronic diseases such as Alzheimer’s and MS. Its effects depend on context, isoforms, and receptor interactions, making it a complex but promising biomarker [27]. Nevertheless, it should be noted that other studies have reported a correlation between serum and cerebrospinal fluid levels [26]. Therefore, this discrepancy in our study might represent a methodological limitation that needs to be considered in the future. While serum NFL levels might reflect central production, OPN in serum and CSF might reflect different cellular sources [49]. Recent evidence suggests a correlation of OPN with white matter [50] and cortical atrophy [31]. High levels of OPN in the CSF of MS patients was recently linked to a reduced brain volume and enlarged ventricles over a decade, but no relationship was observed with peripheral OPN levels, suggesting a key role of central nervous system-localized inflammation, mediated by OPN, in driving long-term brain damage [30]. Finally, NFL and CSF OPN identified patients with a worse prognosis and subsequently treated with highly effective DMTs.

Our study presents some limitations. The main limitations include the small sample size with only three progressive patients and the absence of a control group of healthy subjects. Therefore, our results need to be considered as exploratory. Moreover, no normative values are present for OPN and NFL evaluated with ELLA. Comparison studies with NFL SIMOA revealed differences, with concentrations slightly higher in Ella [15,51]. On the contrary, measuring t-Tau and p-tau with Lumipulse is validated for clinical practice in the search of cognitive impairment: t-Tau higher 410 pg/mL and p-Tau higher than 60 pg/mL are considered abnormal. Only one patient showed high t-tau (with normal p-Tau) but cognition screened with the Brief International Cognitive Assessment for MS was normal. Finally, the lack of a control group and an external validation of our thresholds limits the applicability of our results. Another limitation could be the use of the EDSS as a dichotomic variable with a cut-off of 2.5 in some analyses. However, this was based on the aim of this study to identify patients at risk of early irreversible disability, considering the limitation of higher EDSS scores, which are heavily influenced by ambulation. Finally, more accurate MRI analysis, such as volume lesion load and cortical and sub-cortical atrophy, is needed to confirm our findings, as well as the collection of longitudinal prospective data.

## 5. Conclusions

In conclusion, our data support the presence of both inflammation and axonal loss since the earliest stages of MS. The combined use of CSF and serum biomarkers, particularly neurofilament light chain (NFL) and osteopontin (OPN), might offer valuable insights into the pathological processes underlying early disability. NFL reflects axonal damage and has emerged as one of the most robust biomarkers in MS, while OPN, although not cell-specific, is consistently associated with inflammatory activity within the central nervous system. Importantly, the correlation between these fluid biomarkers and clinical parameters such as EDSS score at diagnosis suggests their potential utility in stratifying patients at higher risk of early disease progression. The ability to identify such patients at the time of diagnosis is crucial, as early therapeutic intervention with high-efficacy treatments may alter the long-term disease trajectory and improve outcomes. Our findings reinforce the concept that a multidimensional biomarker-based approach, integrating measures of both neurodegeneration and inflammation, could possibly enhance clinical decision-making, especially in the context of personalized treatment strategies. While these data are preliminary and exploratory and require longitudinal and repeated validation, they provide a first step for the development of more accurate prognostic tools in MS.

## Figures and Tables

**Table 1:** Clinical–demographic features, disability scores, and biomarker values.

**Characteristics**		***n*/60, (%)**
**Gender**	Female	38 (63%)
**MS type**	Relapsing–remitting	53 (89%)
	Clinically isolated syndrome	2 (3%)
	Radiologically isolated syndrome	2 (3%)
	Secondary progressive	3 (5%)
		**Mean ± SD (years)**
**Age**	Onset	33.0 ± 11.0
	Diagnosis	36.5 ± 10.7
		**Mean, Median, IQR**
**EDSS**	At diagnosis	1.7, 1.5, 1–2.5
**Dd from onset to baseline**		**Mean ± SD** 5.0 ± 7.2
**Brain white matter lesion load**	High lesion load (≥10)	33 (55%)
	Low lesion load (<10)	27 (45%)
**Contrast enhancement (gd+)**	Absent	25 (42%)
	Present	38 (58%)
**Spinal lesions**	Absent	19 (32%)
	Present	41 (68%)
**Onset**	Optic neuritis	17 (29%)
	Sensory/motor	16 (26%)
	Brainstem/cerebellar	10 (17%)
	Spinal	14 (23%)
	Multifocal	1 (2%)
	Radiologically isolated syndrome	2 (3%)
**DMTs**	H-E	16 (27%)
	M-E *	44 (73%)
		**Mean ± SD (pg/mL)**
**Biomarkers**	CSF T-tau	215 ± 79.4
	CSF p-Tau	26.5 ± 7.9
	CSF NFL	2487 ± 4804
	Serum NFL	34.6 ± 24.6
	CSF OPN	174,013 ± 192,495
	Serum OPN	49,329 ± 30,418

Abbreviations: CSF, cerebrospinal fluid; DD, disease duration; SD, standard deviation; EDSS, expanded disability status scale; IQR, interquartile range; CSF, cerebrospinal fluid; NFL, neurofilament light chain; LL, lesion load; DMTs, disease-modifying therapies; H-E, high efficacy; M-E, moderate efficacy; * moderate efficacy + patients who did not start any DMT.

**Table 2:** Univariate analysis between CSF and serum biomarkers at diagnosis.

	CSF NFL	Serum NFL	CSF T-tau	CSF p-Tau	CSF OPN	Serum OPN
**CSF NFL**	-	0.8	0.45	0.13	0.08	0.25
		***p* < *0.0001***	***p = 0.0004***	*p* = 0.3	*p* = 0.5	*p* = 0.05
	**Serum NFL**	-	0.29	−0.03	−0.08	0.43
			***p = 0.02***	*p* = 0.7	*p* = 0.5	***p = 0.0005***
		**CSF T-tau**	-	0.76	0.26	0.06
				***p < 0.0001***	***p* = 0.04**	*p =* 0.6
			**CSF p-Tau**	-	0.30	−0.18
					***p* = *0.01***	*p* = 0.1
				**CSF OPN**	-	−0.15
						*p* = 0.2
					**Serum OPN**	-

CSF biomarker values from MS patients were analyzed for inter-correlations. Several positive correlations were found. The values in each box correspond to the Spearman correlation coefficient (top) and *p*-value (bottom). Values in bold were significant at *p* < 0.05. Abbreviations: CSF: cerebrospinal fluid; NFL: neurofilaments; t-tau: total tau; p-tau: phosphorylated tau; OPN: osteopontin.

**Table 3:** Biomarker comparisons of patients were stratified by MRI, demographic, and MS features.

		**Total Tau**		**p-Tau**	
**T0**		**Mean ± SD**	***p*-Value**	**Mean ± SD**	***p*-Value**
**Brain white matter lesion load**	High lesion load (≥10)	216.7 ± 83.9	0.9	26.7 ± 7.6	0.8
	Low lesion load (<10)	212.9 ± 74.9		26.3 ± 8.5	
**Spinal lesions**	Present	214.0 ± 79.19	0.8	26.9 ± 8.6	0.6
	Absent	219.7 ± 82.9		25.5 ± 6.6	
**Gd+**	Present	218.9 ± 86.7	0.9	26.4 ± 7.9	0.8
	Absent	208.6 ± 67.2		26.7 ± 8.1	
**MS phenotype**	RR	213.5 ± 79.18	0.6	26.2 ± 7.6	0.8
	CIS	174 ± 63.6		23.2 ± 6.9	
	RIS	265.5 ± 119.5		28 ± 5.9	
	SP	235 ± 94		33.4 ± 15.6	
**Sex**	Male	236.1 ± 97.5	0.2	29.5 ± 9.9	0.08
	Female	202.7 ± 65		24.8 ± 6.1	
**Onset**	Optic neuritis	211 ± 72.7	0.6	26 ± 7.6	0.4
	Sensory/motor	199.9 ± 79.9		23.4 ± 7.8	
	Brainstem/cerebellar	244.3 ± 115.9		30.9 ± 8.9	
	Spinal	211.3 ± 53.3		27.3 ± 7.8	
	Multifocal	179 ± 0		26 ± 0	
	RIS	265.5 ± 119.5		28 ± 5.9	
**DMTs**	H-E	229 ± 104.9	0.7	24.4 ± 5.5	0.2
	M-E *	209.8 ± 68.6		27.3 ± 8.6	
		**CSF NFL**		**Serum NFL**	
		**Mean ± SD**	***p*-Value**	**Mean ± SD**	***p*-Value**
**Brain white matter lesion load**	High lesion load (≥10)	2742 ± 6092	0.3	36.2 ± 26.2	0.3
	Low lesion load (<10)	2176 ± 2551		32.5 ± 22.7	
**Spinal Lesions**	Present	2573 ± 5640	0.8	31.8 ± 21.7	0.2
	Absent	2403 ± 2549		41.1 ± 29.6	
**Gd+**	Present	3270 ± 5964	***0.01***	39.4 ± 29.2	***0.04***
	Absent	1228 ± 994.7		26.8 ± 11.3	
**MS phenotype**	RR	2701 ± 5075	0.4	35.77 ± 25.86	0.7
	CIS	541.5 ± 86.9		22.1 ± 6.8	
	RIS	1207 ± 907.9		29.3 ± 12.3	
	SP	861.7 ± 91.5		25.1 ± 2.3	
**Sex**	Male	3372 ± 7447	0.7	35.23 ± 28.06	0.8
	Female	1974 ± 2142		34.19 ± 22.7	
**Onset**	Optic neuritis	1361 ± 911	0.1	30.2 ± 10.1	0.3
	Sensory/motor	1771 ± 2408		31.59 ± 27.09	
	Brainstem/cerebellar	5108 ± 10,850		42.42 ± 37.59	
	Spinal	2603 ± 2332		32.83 ± 15.17	
	Multifocal	7812 ± 0		112 ± 0	
	RIS	1207 ± 907.9		29.3 ± 12.3	
**DMTs**	H-E	4383 ± 8633	***0.049***	45.2 ± 36.7	0.1
	L-E *	1797 ± 1960		30.7 ± 17.3	
		**CSF OPN**		**Serum OPN**	
**T0**		**Mean ± SD**	***p*-Value**	**Mean ± SD**	***p*-Value**
**Brain white matter lesion load**	High lesion load (≥10)	186,836 ± 203,762	0.6	46,239 ± 264,141	0.5
	Low lesion load (<10)	158,340 ± 180,335		53,106 ± 34,842	
**Spinal lesions**	Present	188,053 ± 210,440	0.6	47,358 ± 28,398	0.4
	Absent	143,714 ± 147,058		53,583 ± 34,824	
**Gd+**	Present	199,157 ± 222,831	0.2	46,226 ± 23,619	0.8
	Absent	133,563 ± 123,884		54,322 ± 39,068	
**MS phenotype**	RR	175,163 ± 195,633	0.0508	50,249 ± 32,030	0.8
	CIS	27,988 ± 34,892		34,401 ± 9568	
	RIS	58,773 ± 9643		51,225 ± 11,522	
	SP	327,868 ± 164,976		41,769 ± 13,186	
**Sex**	Male	230,248 ± 210,723	***0.01***	53,520 ± 32,932	0.3
	Female	141,455 ± 175,827		46,903 ± 29,041	
**Onset**	Optic neuritis	148,648 ± 153,565	0.05	54,437 ± 31,892	0.8
	Sensory/motor	223,811 ± 229,099		44,000 ± 18,341	
	Brainstem/cerebellar	126,245 ± 154,789		49,996 ± 42,790	
	Spinal	191,176 ± 230,598		43,593 ± 28,590	
	Multifocal	276,307 ± 0		117,614 ± 0	
	RIS	58,773 ± 9643		51,225 ± 11,522	
**DMTs**	H-E	206,384 ± 249,459	0.7	44,912 ± 27,888	0.5
	M-E *	162,241 ± 169,121		50,935 ± 31,438	

Abbreviations: Gd+, gadolinium-enhancing lesion; SD, standard deviation; EDSS, expanded disability status scale; IQR, interquartile range; CSF, cerebrospinal fluid; NFL, neurofilament light chain; RR, relapsing–remitting; SP, secondary progressive; MS, multiple sclerosis; DMTs, disease-modifying therapies; H-E, high efficacy; M-E, moderate efficacy; * moderate efficacy + patients who did not start any DMT; CIS, clinically isolated syndrome; RIS, radiologically isolated syndrome.

## Data Availability

The original contributions presented in this study are included in the article/Supplementary Material. Further inquiries can be directed to the corresponding author.

## Supplementary Materials

The following supporting information can be downloaded at: https://www.mdpi.com/article/10.3390/neurolint17070110/s1, Table S1: Internal validation of our biomarker thresholds. Table S2: Multiple Regression analyses 1; Table S3: Multiple Regression analyses 2.

## Abbreviations

The following abbreviations are used in this manuscript:

AUC	area under the curve
CIS	clinically isolated syndrome
CSF	cerebrospinal fluid
Dd	disease duration
DMTs	disease-modifying therapies
EDSS	expanded disability status scale
Gd	gadolinium-enhancing
HE	highly effective
IQR	interquartile range
ME	moderate-efficacy
MS	multiple sclerosis
NFL	neurofilament light chain
OPN	osteopontin
PMS	progressive MS
p-Tau	phosporilated tau
RIS	radiologically isolated syndrome
ROC	receiver operating characteristic
RR	relapsing–remitting
SD	standard deviation
SIMOA	single-molecule assay
t-Tau	total tau
