# Supplementary material for: The Association of Axonal Damage Biomarkers and Osteopontin at Diagnosis Could Be Useful in Newly Diagnosed MS Patients
Source: Neurol Int. 2025 Jul 17;17(7):110. doi: 10.3390/neurolint17070110 (PMC12300847; doi:10.3390/neurolint17070110)
Supplement: Supplementary file 1 [file neurolint-17-00110-s001.zip › neurolint-3683396-supplementary.pdf]

Supplementary Materials

Table S1. Internal validation of our biomarker thresholds. Table S1 reports the ROC curve analysis and Youden's index, on a training subset (obtained by randomly selecting 70% of our original dataset). We confirm results for CSF NFL, CSF OPN, and serum NFL.

Area under the curve

				95% Asymptotic Confidence Interval	
Biomarker	Area	Standard Error	p-value	Lower limit	Upper limit
CSF_NFL	,721	,111	,049	,504	,938
Serum NFL	,737	,131	,036	,481	,993
t-Tau	,700	,109	,077	,487	,913
p-Tau	,534	,117	,766	,304	,763
CSF OPN	,779	,090	,014	,603	,955
Serum OPN	,574	,108	,515	,362	,785

Biomarker	Youden Index (training dataset)	Cut-Off
CSF NFL	0.423	3159 pg/ml
Serum NFL	0.625	54.1pg/ml
CSF OPN	0.465	96846.5 pg/ml

Table S2. Multiple Regression analyses 1

Multiple regression analyses were performed to identify the best predictors of disability at diagnosis (using EDSS). The model includes MRI characteristics, age, disease duration form onset, sex, CSF and serum biomarkers at baseline as independent variables. *Abbreviations:* CSF cerebrospinal fluid, dd : disease duration, df : degrees of freedom, EDSS: expanded disability status score, Gd : gadolinium, SC : spinal cord , SE: standard error, WMLL white matter lesion load.

Model Summary

					Change statistics				
Model	R	R2	R2 adjusted	SE of the estimate	R2 change	F change	df1	df2	Sign. F change
1	,830[a]	,688	,609	,82342	,688	8,651	12	47	,000

a. Predictors: (costant), age, spinal cord, CSF total Tau, gd+ lesions, WMLL, serum OPN, CSF OPN, sex, CSF NFL, p-Tau, serum NFL

Coefficients[a]

Model		Non-standardized coefficients B	Standard Error	Standardized coefficients Beta	t	Sign.	95% CI for B Lower level	Upper level
1	(Costant)	-,561	,731		-,767	,447	-2,033	,910
	CSF NFL	7,291E-5	,000	,266	1,595	,117	,000	,000
	Serum NFL	,014	,010	,260	1,443	,156	-,005	,033
	t-Tau	,002	,003	,099	,582	,564	-,004	,007
	p-tau	-,010	,023	-,062	-,447	,657	-,056	,036
	CSF OPN	1,455E-6	,000	,213	2,266	***,028***	,000	,000
	Serum OPN	-1,119E-6	,000	-,026	-,255	,800	,000	,000
	Age	,011	,012	,093	,955	,345	-,013	,036
	Dd onset	,071	,017	,394	4,200	***,000***	,037	,105
	Sex	-,074	,257	-,027	-,287	,776	-,591	,443
	WMLL	,113	,230	,043	,493	,624	-,349	,575
	SC	,766	,259	,273	2,964	***,005***	,246	1,286
	Gd+	,181	,249	,068	,728	,470	-,320	,682

a. Dependent variable: EDSS at diagnosis

Table S3. Multiple Regression analyses 2

Multiple regression analyses were performed to identify the best predictors of first treatment choice (HE vs LE DMT). The model includes MRI characteristics, age, sex, CSF and serum biomarkers at baseline as independent variables. *Abbreviations:* CSF cerebrospinal fluid, dd: disease duration, df: degrees of freedom, EDSS: expanded disability status score, Gd : gadolinium, SC : spinal cord , SE: standard error, WMLL white matter lesion load.

Model Summary

Model	R	R2	R2 adjusted	SE of the estimate	Change statistics R2 change	F change	df1	df2	Sign. F change
2	,701[a]	,491	,348	,360	,491	3,417	13	46	,001

a Predictors: (costant), age, spinal cord, CSF total Tau, gd+ lesions, WMLL, serum OPN, CSF OPN, sex, CSF NFL, p-Tau, serum NFL

Coefficients[a]

Model	Non-standardized coefficients	Standardized coefficients	t	Sign.	95% CI for B

		B	Standard Error	Beta			Lower level	Upper level
1	(Costant)	,434	,322		1,346	,185	-,215	1,082
	NFL CSF	-1,556E-5	,000	-,168	-,758	,453	,000	,000
	NFL serum	,003	,004	,155	,654	,517	-,006	,012
	t-Tau	,001	,001	,109	,490	,627	-,002	,003
	p-tau	-,019	,010	-,340	-1,885	,066	-,039	,001
	CSF OPN	4,499E-8	,000	,019	,152	,880	,000	,000
	Serum OPN	-3,195E-6	,000	-,218	-1,661	,103	,000	,000
	Age	-,008	,005	-,181	-1,419	,163	-,018	,003
	Dd onset	-,011	,009	-,179	-1,264	,213	-,028	,006
	Sex	,147	,112	,160	1,304	,199	-,080	,373
	WMLL	,328	,101	,369	3,261	***,002***	,126	,531
	SC	-,023	,123	-,024	-,187	,853	-,271	,225
	Gd+	-,064	,110	-,070	-,580	,565	-,284	,157
	Edss	,171	,064	,505	2,681	***,010***	,043	,300

a. dependent variable: first treatment choice (HE vs LE DMT)
